# Discontinuing absorbent pants in children with bedwetting: a randomized controlled trial

**DOI:** 10.1007/s00431-024-05502-w

**Published:** 2024-03-12

**Authors:** Anders Breinbjerg, Konstantinos Kamperis, Kristina Thorsteinsson, Cecilie Siggaard Jørgensen, Lien Dossche, Juliette Rayner, Jin Zhang, Debora Garcia Rodrigues, Luise Borch, Søren Hagstrøm, Serdar Tekgül, Johan Vande Walle, Søren Rittig

**Affiliations:** 1 Department of Pediatrics, Aarhus University Hospital, Palle Juul-Jensens blvd. 99, 8200 Aarhus N, Denmark; 2 Department of Clinical Medicine, Aarhus University, Aarhus, Denmark; 3 Department of Pediatrics and Adolescent Medicine, Aalborg University Hospital, Aalborg, Denmark; 4 Department of Pediatric Nephrology, ERKNET center, Ghent University Hospital, Ghent, Belgium; 5 ERIC, The Children’s Bowel and Bladder Charity, 36 Old School House, Kingswood Foundation, Brittania Rd, Bristol, BS15 8DB UK; 6 Global Product Safety, Stewardship & Medical Affairs, Kimberly-Clark Corporation, Tadworth, UK; 7 Department of Pediatric and Adolescent Medicine, Gødstrup Hospital, Herning, Denmark and NIDO, Centre for Research and Education, Gødstrup Hospital, Herning, Denmark; 8 Division of Pediatric Urology, Department of Urology, Medical School, Hacettepe University, Ankara, Turkey

**Keywords:** Nocturnal enuresis, Nappy, Nappies, Diaper, Toilet training

## Abstract

The objective of this study is to examine the effect of discontinuing wearing protective garments (absorbent pyjama pants — APP) in children with severe childhood nocturnal enuresis (NE). The study employs a multicenter, parallel, randomized controlled trial. Following a 4-week run-in period, participants were randomly allocated in a 2:1 group allocation to discontinue or continue using APP. The research was conducted across seven European pediatric incontinence centers. The study included treatment-naïve children aged 4–8 years with severe (7/7 wet nights per week) mono-symptomatic NE, who had used nighttime protection for at least 6 months prior to the study. The study consisted of a 4-week run-in period (± 7 days), where all children slept wearing APP (DryNites^®^). At week 4 (± 7 days), if meeting randomization criteria (7/7 wet nights during the last week of run-in), participants were randomized to continue to sleep in APP or to discontinue their use for a further 4 weeks, with the option of another 4 weeks in the extension period. The primary outcome was the difference between groups of wet nights during the last week of intervention. Quality of life (QoL) and sleep were secondary endpoints. In total, 105 children (43 girls and 62 boys, mean age 5.6 years [SD 1.13]) were randomized (no-pants group *n* = 70, pants group *n* = 35). Fifteen children (21%) in the no-pants group discontinued early due to stress related to the intervention. Children in the no-pants group experienced fewer wet nights compared to the pants group during the last week (difference 2.3 nights, 95% CI 1.54–3.08; *p* < 0.0001). In the no-pants group, 20% responded to the intervention, of whom 13% had a full response. Clinical improvement was detected within 2 weeks. Sleep and QoL were reported as negatively affected by APP discontinuation in the extension period but not in the core period.

*    Conclusion*: A ~ 10% complete resolution rate was associated with discontinuing APP. While statistically significant, the clinical relevance is debatable, and the intervention should be tried only if the family is motivated. Response was detectable within 2 weeks. Discontinuing APP for 4–8 weeks was reported to negatively affect QoL and sleep quality. No severe side effects were seen.

*Trial registration*: Clinicaltrials.gov Identifier: NCT04620356; date registered: September 23, 2020. Registered under the name: “Effect of Use of DryNites Absorbent Pyjama Pants on the Rate of Spontaneous Resolution of Paediatric Nocturnal Enuresis (NE).”

**Supplementary Information:**

The online version contains supplementary material available at 10.1007/s00431-024-05502-w.

**Table:** 

**What is Known?** *• APP has been hypothesized to delay time until spontaneous resolution of bedwetting, but no prospective randomized evidence is available regarding if APP use sustains symptoms.* *• No evidence whether discontinuation of APP can lead to dryness or impact sleep quality and QoL.*	
**What is New?** *• Thirteen percent became dry after removing APP for up to 8 weeks and initial response was detectable within 2 weeks.* *• Even though prolonged discontinuation was reported to lead to sleep disturbances and rediced QoL, still our results suggest trying 2 weeks of the intervention if the family is motivated, prior to engaging established treatments for nocturnal enuresis. *	

## Introduction

### Background

Nocturnal enuresis (NE) is a common disorder, affecting approximately 7–10% of all 7-year old children [1]. The condition can be distressing [2], negatively affecting self-esteem, and it can lead to bullying and social withdrawal [3]. It thus demands attention and treatment.

Common first-line treatments for NE involve behavioral modifications, standard urotherapy [4], desmopressin and/or conditioning with an enuresis alarm, along with treatment of potential comorbidities [5]. The efficacy of alarm treatment and desmopressin is well established [6, 7], whereas standard urotherapy seems ineffective at least for the monosymptomatic form of NE [8, 9].

The use of absorbent pyjama pants (APP) in coping with NE has been intensely debated in recent years, as some research has suggested that use of APP may prolong the time until spontaneous resolution of the condition [10, 11], but findings are prone to bias, especially due to concurrent toilet training (TT) practices. On the other hand, use of APP is an effective way to reduce the impact of NE and to improve sleep [12].

Guideline recommendations on managing NE are abundant; however, advice on using APP remains limited [13, 14]. Current guidelines suggest periods sleeping without APP, but these recommendations are not scientifically robust [15, 16]. Furthermore, although undocumented, in many countries, healthcare professionals are against using APP to manage NE. The area is controversial, and prospective studies are needed. The intervention of removing APP in children with NE is simple and could be carried out before seeking more established treatments; hence, investigation in children below the age of 6 years might be reasonable.

Until now, no prospective randomized trials have assessed whether use of APP sustains NE symptoms and severity, or whether removal of APP can lead to dryness. Furthermore, no evidence is available regarding the impact of removal of APP on sleep quality and QoL measures.

### Objectives and endpoints

We aimed to investigate the effect of continuing or discontinuing use of APP (DryNites^®^) on NE frequency in children aged 4–8 years with severe primary monosymptomatic NE. We further aimed to investigate quality of life (QoL) and sleep measures.

## Methods

### Trial design

**Fig. 1:**
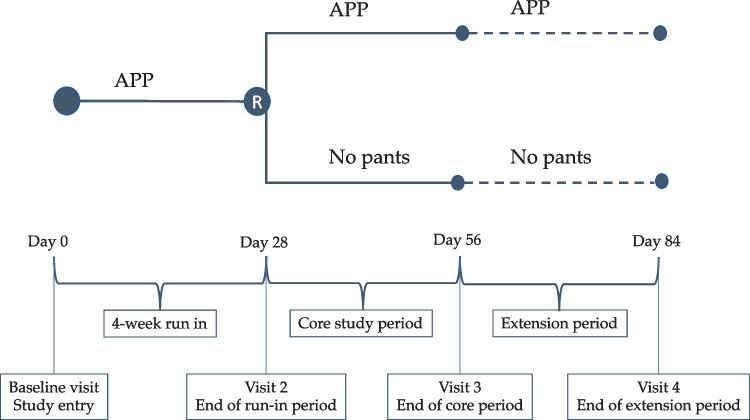
Flow of the study. Initially, all participants underwent 4 weeks (± 7 days) in the run-in period. If eligible (wet 7/7 days), the children were randomized to continue or discontinue the use of APP during sleep in the core period. If motivated, the families could choose to enter the extension period for 4 weeks more after the core period. APP, absorbent pyjama pants

An 8-week, investigator initiated, randomized, controlled, open-label, multicenter, two-arm, parallel-group phase IV trial was conducted to assess the effect of discontinuing (no-pants group) versus continuing (pants group) the use of APP on NE symptoms in children with severe NE, with an optional 4-week extension period (Fig. 1).

Participants needed to have 7/7 wet nights in the last week of the run-in period. Eligible participants were then randomly assigned (2:1) to either discontinuation or continuation of APP for the 4-week core intervention period. Full inclusion and exclusion criteria are defined in Table 1. Randomization was performed electronically. Children included were strictly monosymptomatic, as defined by the ICCS [5].

**Table 1:** Inclusion and exclusion criteria

**Inclusion criteria**
The following criteria must be met for the participant to be enrolled in the study
1. Patient aged between 4 and 8 years at the time of enrolment
2. Have a clinical diagnosis of monosymptomatic primary NE
3. Have been dry in the day for ≥ 6 months prior to enrolment
4. Have on average no more than one dry night per month during the past 6 months at enrolment
5. Using absorbent pants/nappies to manage NE for at least 6 months prior to enrolment
6. Have an informed consent form (ICF) signed by their parent(s)/carer(s)
7. For randomization: have NE 7 nights per week over week 4/last week of the run-in period

**Exclusion criteria**
Patients meeting ANY of the following criteria are not eligible for participation
1. Children in foster/court care
2. Have implemented any previous intervention to address NE (use of prescribed alarm schedule, desmopressin, imipramine, anticholinergics), or withdrawal of pants/nappies for > 7 days in the previous 6 months
3. Have secondary NE
4. Have wetting in the day
5. Have fecal soiling
6. Have known urinary tract disease
7. Have diabetes
8. Receive any regular intake of medication
9. Have a known developmental/neurological disorder
10. Have links to Kimberly-Clark of any kind (including family relations employed by Kimberly-Clark, holding stocks or shares in Kimberly-Clark)

*NE* nocturnal enuresis, *ICF* informed consent form

Protocol amendments occurred due to Covid-19 restrictions, permitting remote assessments. Study findings are reported in accordance with the CONSORT 2010 statement [17]. The study was approved by the regional ethical committees in Denmark, Belgium, and the UK (see Supplementary for details) and was carried out according to the declaration of Helsinki. Prior to enrollment, both written and oral informed consent, by both parents, were obtained.

### Interventions

Participants fulfilling the inclusion criteria entered a 4-week run-in period during which they slept wearing the intervention APP. The children sleeping without APP were permitted to sleep with an absorbent bed mat instead. The DryNites^®^ disposable APP was used. APP and bed mats for the study period were supplied to the families by the sponsor. No behavioral changes such as fluid restriction or lifting the child to the toilet when the parents went to bed were allowed in either group. After completing the core intervention period, participants were invited to take part in a 4-week extension period, during which they would remain on their randomly assigned treatment.

### Outcomes

Demographic data was collected at enrollment (height, weight, body mass index [BMI]). The primary outcome was the average number of wet nights in the last week of the 4-week intervention period, evaluated using an electronic diary.

The secondary outcomes of quality-of-life (QoL) and sleep parameters were assessed using the following validated questionnaires: Pediatric Incontinence Questionnaire (PinQ) [18], World Health Organization Quality of Life Brief Version [19], Pediatric Daytime Sleepiness Scale (PDSS) [20], and Checklist Individual Strength [21].

Study outcomes were recorded using an electronic diary and embedded into an online electronic data capture system, which was accessible from the parents’ own devices. Originally, we aimed to analyze the data in two age groups: 4–5 and 6–8 years. However, it proved hard to include children older than 5 years, as the families had already begun or wanted to try more established treatments first. Hence, we changed this aim, and we present data here on all children, 4–8 years of age, combined. Questionnaires were completed jointly by children and parents.

### Statistical methods

Analyses were performed with guidance from the IQvia biostatistician team and the core facility Biostatistical Advisory Service at Aarhus University, Aarhus, Denmark.

The primary outcome was assessed by comparing the last 7 days of intervention between groups, and a risk difference was calculated. To handle missing data, imputations, sensitivity and intention-to-treat analyses were performed (see Supplementary for details).

Secondary outcomes were assessed by comparing mean scores and individual items between groups at the different timepoints.

Post hoc analyses were performed for both primary and secondary outcomes, including responder group analyses performed according to the International Children’s Continence Society recommendations [22]. Time-to-effect and time-to-dropout were evaluated using time-to-event plots. For full statistical methods, see the Supplementary.

## Results

### Participant flow and recruitment

**Fig. 2:**
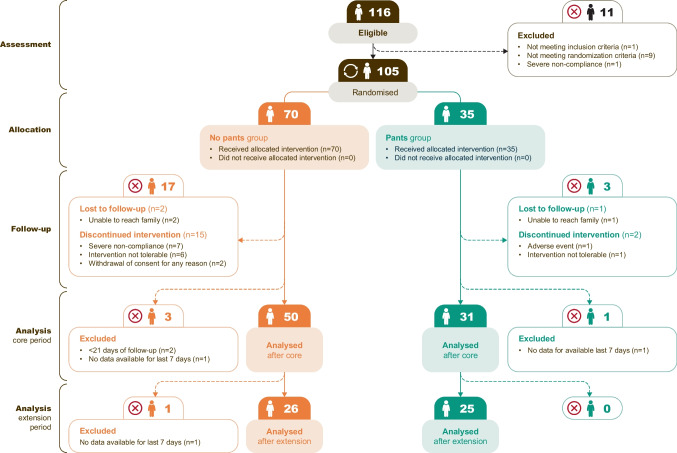
Overview of children enrolled, randomized, allocation, and reasons for discontinuation following the CONSORT flowchart recommendations

Between February 21, 2020, and October 2022, 116 patients were assessed for eligibility, 105 of whom were randomized (Fig. 2). End of follow-up was 2022. After run-in, 70 children were allocated to the no-pants group and 35 were allocated to the pants group. Demographics can be seen in Supplementary Table 1. In total, 85 and 52 children completed the core and extension periods, respectively. No participants experienced severe adverse events.

### Primary outcome and estimations of response

The difference in the average number of wet nights between groups after the core and extension periods can be seen in Table 2. In summary, the analyses indicated a significant difference between groups at both the end of the core and the end of the extension period, favoring a lower frequency of enuresis in the no-pants group. In the sensitivity analysis imputed from participant data, the risk difference marginal mean was 0.33 (95% CI 0.22-0.44), which is equivalent to a difference of 2.31 (95% CI 1.54–3.08) wet nights less in the no-pants group at the end of the core period (*p* < 0.0001).

**Table 2:** Primary outcome results

**Last 7 days analysis — crude**^**a**^								
	**End of core period (*n* = 81)**				**End of extension period (*n* = 51)**			
**Group**	**Estimated marginal mean**	**SE**	**95% CI**	***p-*value**	**Estimated marginal mean**	**SE**	**95% CI**	***p-*value**
Pants	0.96	0.015	0.93–0.99		0.92	0.036	0.85–0.99	
No-pants	0.64	0.052	0.54–0.74		0.47	0.079	0.31–0.62	

**Risk difference**	**Estimate**	**SE**	**95% CI**		**Estimate**	**SE**	**95% CI**	
Pants — no-pants	0.32	0.054	0.22–0.43	< 0.0001	0.45	0.087	0.28–0.62	< 0.0001

**Last 7 days analysis — sensitivity analysis 1 (all missing values as dry nights)**								
**Group**	**Estimated marginal mean**	**SE**	**95% CI**	***p-*value**	**Estimated marginal mean**	**SE**	**95% CI**	
Pants	0.81	0.033	0.74–0.87		0.70	0.063	0.57–0.82	
No pants	0.54	0.047	0.45–0.63		0.36	0.072	0.22–0.50	

**Risk difference**	**Estimate**	**SE**	**95% CI**		**Estimate**	**SE**	**95% CI**	
Pants — no-pants	0.27	0.058	0.16–0.38	< 0.0001	0.34	0.096	0.15–0.52	0.0005

**Last 7 days analysis — sensitivity analysis 2 (all missing values as wet nights)**								
**Group**	**Estimated marginal mean**	**SE**	**95% CI**	***p-*value**	**Estimated marginal mean**	**SE**	**95% CI**	
Pants	0.97	0.013	0.94–0.99		0.94	0.026	0.87–0.99	
No pants	0.68	0.044	0.61–0.78		0.58	0.058	0.47–0–70	

**Risk difference**	**Estimate**	**SE**	**95% CI**		**Estimate**	**SE**	**95% CI**	
Pants — no-pants	0.27	0.046	0.18–0.36	< 0.0001	0.36	0.064	0.23–0.48	< 0.0001

**Last 7 days analysis — sensitivity analysis 3 (imputed from participant data)**								
**Group**	**Estimated marginal mean**	**SE**	**95% CI**	***p-*value**	**Estimated marginal mean**	**SE**	**95% CI**	
Pants	0.96	0.016	0.93–0.99		0.86	0.056	0.75–0.97	
No-pants	0.63	0.051	0.53–0.73		0.42	0.079	0.27–0.58	

**Risk difference**	**Estimate**	**SE**	**95% CI**		**Estimate**	**SE**	**95% CI**	
Pants — no-pants	0.33	0.053	0.22–0.44	< 0.0001	0.44	0.097	0.25–0.63	< 0.0001

**Last 7 days analysis — sensitivity analysis 4 (intention-to-treat, all randomized patients included, *n* = 105)**								
**Group**	**Estimated marginal mean**	**SE**	**95% CI**	***p-*value**				
Pants	0.95	0.018	0.91–0.98					
No-pants	0.72	0.040	0.64–0.79					

**Risk difference**	**Estimate**	**SE**	**95% CI**					
Pants — no-pants	0.24	0.044	0.14–0.32	< 0.0001				

*SE* standard error, *CI* confidence interval

^a^Approximately 25% missing data for the last 7 days

**Fig. 3:**
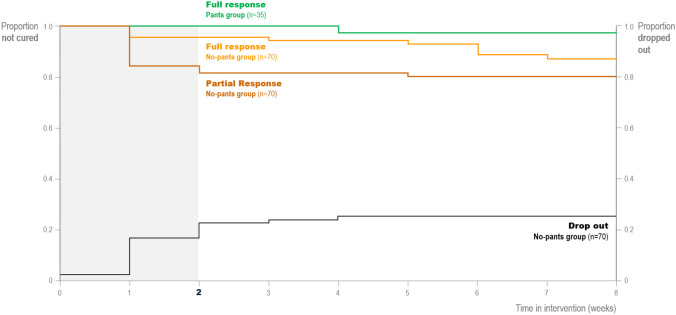
The different trajectories depict the number of full (orange) and partial (brown) responders in the no pants and the full responders (green) in the pants group. The *x*-axis represents the time in weeks. The *y*-axis to the left shows the proportion of children not cured. The black trajectory depicts the dropout rate, and the y-axis to the right shows the proportion of children who dropped out. Assessments of status were done at the end of each week. If all seven nights in a week were dry, then the child was a full responder for that week; if they had ≥ four dry nights, the child was a partial responder; and if they left the study, they were a dropout

Post hoc responder group results can be seen in Table 3. Results indicated a significant improvement in the no-pants group compared with the pants group in children who completed the study (*p* = 0.016), but this was not significant when using an intention-to-treat analysis (*p* = 0.056). Half of the nine full responders (n = 5) achieved a full response during the extension period (Fig. 3).

**Table 3:** Response groups analysis results

**Per protocol** **Children who completed the study**		
**Response group**	**No-pants group (*n* = 50)**	**Pants group (*n* = 32)**
Full response^a^ (%)	9 (18)	1 (3)
Partial response^b^ (%)	5 (10)	0 (0)
No response^c^ (%)	36 (72)	31 (97)
Chi-square value	8.21, 2 degrees of freedom, *p* = 0.0164	

**Intention to treat** **All randomized children, missing children (dropouts, non-compliers, non-responders stopping at visit 3) imputed with a probability of 0.961 of being wet**		
**Response group**	**No-pants group (*n* = 70)**	**Pants group (*n* = 35)**
Full response^a^ (%)	9 (13)	1 (3)
Partial response^b^ (%)	5 (7)	0 (0)
No response^c^ (%)	56 (80)	34 (97)
Chi-square value	5.75, 2 degrees of freedom, *p* = 0.056	

^a^100% improvement of symptoms

^b^50–99% improvement of symptoms

^c^< 50% improvement of symptoms

The time to effect and time to dropout in the intention-to-treat population (*n* = 105) is shown in Fig. 3. Approximately 20% of children experienced a reduction in enuresis frequency of more than 50% compared with baseline in the no-pants group (*n* = 70); most of these children had a visible effect after week 1 of intervention. Approximately 20% of children in the no-pants group could not cope with the intervention and discontinued within the first 3 weeks.

### Secondary endpoints and estimations of response

Per-protocol and post hoc analyses were conducted, with mean (SD) and least-squares mean (standard error) values, along with the mixed models for repeated measures (MMRM) models of the included questionnaires — PinQ, PDSS, World Health Organization Quality of Life Brief Version, and CIS (data not shown).

Overall, no findings were significant in the core analysis set, including all randomized participants, in all four questionnaires. Looking to the extension analysis set, only including participants who completed the core and the extension period, the comparison between groups of the total score in the PinQ, between visit 2 and at visit 3, was significantly higher in the no-pants group (*p* = 0.029). This tendency was also seen at visit 2 between the groups; however, this finding was not statistically significant. The MMRM model comparing total scores between groups at visit 3 showed a significantly higher LS mean in the no-pants group (17.7 (SE 2.2) vs 10.4 (SE 2.4), *p* = 0.029). The same became evident in the extrinsic and intrinsic items at visit 3 of the PinQ (*p* = 0.011 and *p* = 0.044, respectively). The overall MMRM model including data from all visits was significant in the extrinsic score only, comparing no-pants to the pants group (LS mean 4.9 (SE 0.6) vs 2.9 (SE 0.6), *p* = 0.019). These findings suggest a higher impact of the NE in the no-pants group.

In post hoc analysis of PinQ data, no significant difference was found in the total score. In item 11: “*I wake up during my sleep because of my bladder problem*,” the non-responders in the no-pants group had a mean score of 1.9 (SD 1.32) versus 1.11 (SD 1.10) in the pants group (*p* < 0.05), suggesting children were awake for more of their NE episodes in the no-pants group. In a post hoc analysis of the PDSS questionnaire, in item 4: “How often are you ever tired and grumpy during the day?,” the mean value for the non-responders in the no-pants group was 1.63 (SD 0.81) compared with 1.17 (SD 0.60) in the pants group (*p* = 0.019), suggesting a significantly higher level of tiredness/grumpiness during the day. In a post hoc analysis, the Checklist Individual Strength questionnaire suggested that items 1: “I feel tired,” 7: “I do quite a lot within a day,” and 12: “I feel rested,” seems more negatively affected in the no-pants group, but not significantly.

## Discussion

In this study, children sleeping without APP had a statistically significantly greater reduction in NE frequency compared with those who continued to use APP. In the no-pants group, 20% of children had ≥ 50% reduction of enuresis frequency. Complete resolution of NE was achieved by 13% of the no-pants group, compared with 3% in the pants group. Response, if any, was seen early in the intervention. QoL and sleep was reported by the families to be negatively affected by the intervention. To our knowledge, this is the first randomized controlled trial investigating the effect of removing absorbent pants on the severity of childhood NE.

The prevalence of NE has been reported to be increasing, especially in countries where the use of APP has recently been adopted, like China [10], and a discussion of whether or not APP have affected this increase, together with a change of practice in TT, is ongoing [23]. Current evidence on the effect of use of APP on incontinence is based upon cross-sectional and retrospective questionnaire studies, and the body of evidence from prospective studies is limited to smaller case studies [24, 25], both of which suggested a positive effect on incontinence frequency of not using APP. In these studies, the intervention of not using APP was combined with TT, and conclusions on the impact of isolated APP removal are unclear. One large prospective trial performed by van Dommelen et al*.* [26] investigated the effect of different behavioral interventions in children with NE and identified APP use as a significant risk factor for not responding to the interventions after 6 months of training. Again, conclusions were hard to draw as no randomization was performed regarding APP use. In summary, no prospective evidence has supported the hypothesis that use of APP prolongs NE, as many factors, especially TT practices, are hard to adjust for. The results of the present randomized controlled study suggesting a significantly lower number of wet nights after the intervention are in support of the hypothesis generated from these case and epidemiological studies.

We found a statistically significant reduction in frequency of wet nights of approximately 2 nights per week after 4 weeks of intervention, and approximately 13% of children had a full response after the study period. The efficacy of other, more established first-line treatments for NE (i.e., desmopressin and the enuresis alarm) is markedly higher; the proportions achieving full response may reach 50–70% [27, 28]. It is important to stress that the response rates in the present study are obtained in children with severe NE. The response rates in children with less severe symptoms might be different. Most children experiencing full and partial response after extension obtained some visible effect during the first or second week of intervention. Half of the nine full responders (*n* = 5) achieved a full response during the extension period. This suggest that children experiencing a partial response during the initial weeks of intervention may expect further improvement if continuing the intervention.

Whether children experiencing dry nights start waking up to go to the toilet or simply postpone bladder emptying until the next morning and why this effect is seen so early during the intervention is worth considering. Sleep studies have suggested both an increased arousal index [29] and others that some enuretic children are awake (on electroencephalogram) when emptying their bladder at night [30]. The mechanism could be similar to enuresis alarm treatment, in which the suggested mode of action is the conditioning of bladder-brain communication either by enabling/training the brain to awaken just before bladder emptying occurs or by suppressing the voiding reflex during sleep. In the present study, no systematic registration of nighttime awakenings was performed. However, six participants in the no pants group with treatment response uniformly described several nights with awakenings and nocturia followed by dry nights with uninterrupted sleep. This mode of developing dryness has also been reported in enuresis alarm treatment (Hagstrøm et al. unpublished data).

The spontaneous resolution rate of NE has been calculated to be approximately 15% per year and seems to be stable throughout childhood [31]. However, children with severe NE seems to have a lower resolution rate [32]. Whether or not the rate of resolution is affected by using APP is debatable and beyond the scope of this study as it would require a much longer observation period. Such a study would be very difficult to perform as it would be unethical to continue an intervention if no effect was observed. Another important point is that one participant in the pants group had spontaneous resolution, emphasizing that children can get dry even when sleeping with APP.

The removal of APP was not tolerable in a substantial number of the families, as approximately 20% discontinued early due to inconvenience and/or anxiety related to the intervention.

Discontinuation rates as high as 60% have been reported with use of the enuresis alarm [33]. Between countries, healthcare professionals’ advice differs regarding APP, spanning from the notion that they may prolong bedwetting [34] to the belief that they are an easy way of coping with NE symptoms until active treatment initiation. Our results suggest that only a small subset of children will benefit from APP removal. We believe that the choice of using APP or not should be the family’s decision after appropriate information on the expected outcomes, advantages, and disadvantages along with their evaluation on other existing treatment options.

The secondary outcome questionnaires used were originally not designed specifically to investigate an intervention such as this, and hence, total scores were not expected to be affected. Instead, when analyzing specific questions of interest, we found several significant associations. Overall, in the extension set, QoL was reported to be negatively affected in the no-pants group, and post hoc analyses suggested that children were less rested and had more awakenings in the no-pants group. If the intervention is seen as a potential treatment for NE, discomfort must be expected, as with the enuresis alarm. However, sleeping without APP for longer periods of time without experiencing improvement might be unwise as the intervention may affect daytime energy levels.

### Limitations

This study suffered from a skewed dropout rate between groups, most likely due to the inconvenience of the intervention, as well as missing data in diaries. We have statistically attempted to adjust and interpret the findings following appropriate methods and believe that the findings are relatively robust.

Questionnaire data at visit 3 were not obtained in the majority of the families who discontinued early, and this is a clear limitation, as we would expect these families to have reported a higher amount of stress compared with families who actually completed the study. This might explain the smaller difference in questionnaire data in the per-protocol analysis. Furthermore, the questionnaires regarding child QoL [18] and sleep [20] used were not validated for children down to the age of 4 years, and hence, interpretation should be made cautiously.

The intervention period could have been longer to enable investigation of long-term effects. However, the intervention period was chosen considering well-being of the participants. Also, there might be a possible selection bias in the enrollment, as all families participating were actively seeking help for NE.

This study investigates only children with a prior use of APP, and hence results, especially secondary outcomes, cannot be extrapolated to children who do not use APP or similar to cope with NE.

The mean ages of the children in this study were 5.6 and 5.4 years of age, in the no-pants and pants groups, respectively. We might speculate that the response rates may be different in older age groups, as maturity and developmental stage could potentially influence the response.

### Generalizability

As several study centers were spread across Europe, we argue that our findings have a high external validity, and generalization to other children in countries with similar healthcare systems and toileting culture seems appropriate, in children with severe NE.

## Conclusion

While statistically significant, the clinical relevance of the modest symptom amelioration achieved by discontinuation is limited, and the decision to attempt the intervention must be based on family motivation. During prolonged (4–8 weeks) discontinuation, unmanaged NE was reported by the families to lower the sleep quality and QoL of caregivers and their children. We propose attempting a 2-week period without APP, continuing if response is obtained. If no response is seen in the families who have used APP prior to the intervention, it is advisable to reinitiate APP use to restore sleep quality and QoL in children and caregivers.

### Supplementary Information

Below is the link to the electronic supplementary material.

Supplementary file1 (DOCX 37 KB)

## Data Availability

Availability of the data underlying this publication will be determined later according to Kimberly-Clark’s commitment to the EFPIA/PhRMA “Principles for responsible clinical trial data sharing”. This pertains to scope, time point and process of data access.As such, Kimberly-Clark commits to sharing upon request from qualified scientific and medical researcher’s patient-level clinical trial data, study-level clinical trial data, and protocols from clinical trials in patients for products approved in the United States (US) and European Union (EU) as necessary for conducting legitimate research. Interested researchers can use andbre@clin.au.dk to request access to anonymized patient-level data and supporting documents from clinical studies to conduct further research that can help advance medical science or improve patient care. Data access will be granted to anonymized patient-level data, protocols and clinical study reports after approval by an independent scientific review panel. Kimberly-Clark is not involved in the decisions made by the independent review panel. Kimberly-Clark will take all necessary measures to ensure that patient privacy is safeguarded.

## Abbreviations

APP: Absorbent pyjama pants
CI: Confidence interval
MMRM: Mixed models for repeated measures
NE: Nocturnal enuresis
PDSS: Pediatric Daytime Sleepiness Scale
PinQ: Pediatric Incontinence Questionnaire
SD: Standard deviation
TT: Toilet training

## References

[1] von Gontard A, Heron J, Joinson C (2011). Family history of nocturnal enuresis and urinary incontinence: results from a large epidemiological study. J Urol.

[2] Van Tijen NM, Messer AP, Namdar Z (1998). Perceived stress of nocturnal enuresis in childhood. Br J Urol.

[3] Hagglof B, Andren O, Bergstrom E, Marklund L, Wendelius M (1998). Self-esteem in children with nocturnal enuresis and urinary incontinence: improvement of self-esteem after treatment. Eur Urol.

[4] Nieuwhof-Leppink AJ, Hussong J, Chase J, Larsson J, Renson C, Hoebeke P, Yang S, von Gontard A (2021). Definitions, indications and practice of urotherapy in children and adolescents: - a standardization document of the International Children’s Continence Society (ICCS). J Pediatr Urol.

[5] Nevéus T, Fonseca E, Franco I, Kawauchi A, Kovacevic L, Nieuwhof-Leppink A, Raes A, Tekgül S, Yang SS, Rittig S (2020). Management and treatment of nocturnal enuresis-an updated standardization document from the International Children’s Continence Society. J Pediatr Urol.

[6] Glazener CM, Evans JH (2000) Desmopressin for nocturnal enuresis in children. The Cochrane database of systematic reviews CD002112. 10.1002/14651858.cd002112 10.1002/14651858.CD002112 PMC8984681 10796860

[7] Glazener CM, Evans JH, Peto RE (2005) Alarm interventions for nocturnal enuresis in children. Cochrane Database Syst Rev (Online):CD002911. PMID: 15846643. 10.1002/14651858.CD002911.pub2 10.1002/14651858.CD002911.pub2 15846643

[8] Borgström M, Bergsten A, Tunebjer M, Hedin Skogman B, Nevéus T (2022). Daytime urotherapy in nocturnal enuresis: a randomised, controlled trial. Arch Dis Child.

[9] Jørgensen CS, Kamperis K, Walle JV, Rittig S, Raes A, Dossche L (2023). The efficacy of standard urotherapy in the treatment of nocturnal enuresis in children: a systematic review. J Pediatr Urol.

[10] Wang XZ, Wen YB, Shang XP, Wang YH, Li YW, Li TF, Li SL, Yang J, Liu YJ, Lou XP, Zhou W, Li X, Zhang JJ, Song CP, Jorgensen CS, Rittig S, Bauer S, Mosiello G, Wang QW, Wen JG (2019). The influence of delay elimination communication on the prevalence of primary nocturnal enuresis-a survey from Mainland China. Neurourol Urodyn.

[11] Breinbjerg A, Rittig S, Kamperis K (2021). Does the development and use of modern disposable diapers affect bladder control? A systematic review. J Pediatr Urol.

[12] Kushnir J, Cohen-Zrubavel V, Kushnir B (2013). Night diapers use and sleep in children with enuresis. Sleep Med.

[13] (2005) Management of primary nocturnal enuresis. Paediatr Child Health 10:611–614. PMID: 19668675, PMCID: PMC2722619. 10.1093/pch/10.10.611 10.1093/pch/10.10.611 PMC2722619 19668675

[14] National Clinical Guideline C (2010) National Institute for Health and Clinical Excellence: guidance. Nocturnal enuresis: the management of bedwetting in children and young people. Royal College of Physicians (UK) Copyright ^©^ 2010, National Clinical Guideline Centre London. PMID: 22031959, Bookshelf ID: NBK62712 22031959

[15] Excellence NIfHaC (2017) Bedwetting in under 19s. https://www.nice.org.uk/guidance/cg111

[16] Schmitt BD (1997) Nocturnal enuresis. Pediatr Rev 18:183–190; quiz 191. PMID: 9167432. 10.1542/pir.18-6-183 10.1542/pir.18-6-183 9167432

[17] Schulz KF, Altman DG, Moher D (2010). CONSORT 2010 statement: updated guidelines for reporting parallel group randomised trials. J Pharmacol Pharmacother.

[18] Bower WF, Sit FK, Bluyssen N, Wong EM, Yeung CK (2006). PinQ: a valid, reliable and reproducible quality-of-life measure in children with bladder dysfunction. J Pediatr Urol.

[19] World Health Organisation WHOQOL-BREF. https://iris.who.int/handle/10665/63529

[20] Drake C, Nickel C, Burduvali E, Roth T, Jefferson C, Pietro B (2003). The pediatric daytime sleepiness scale (PDSS): sleep habits and school outcomes in middle-school children. Sleep.

[21] Vercoulen JH, Swanink CM, Fennis JF, Galama JM, van der Meer JW, Bleijenberg G (1994). Dimensional assessment of chronic fatigue syndrome. J Psychosom Res.

[22] Austin PF, Bauer SB, Bower W, Chase J, Franco I, Hoebeke P, Rittig S, Walle JV, von Gontard A, Wright A, Yang SS, Nevéus T (2016). The standardization of terminology of lower urinary tract function in children and adolescents: update report from the standardization committee of the International Children’s Continence Society. Neurourol Urodyn.

[23] Li X, Wen JG, Shen T, Yang XQ, Peng SX, Wang XZ, Xie H, Wu XD, Du YK (2020). Disposable diaper overuse is associated with primary enuresis in children. Sci Rep.

[24] Tarbox RS, Williams WL, Friman PC (2004). Extended diaper wearing: effects on continence in and out of the diaper. J Appl Behav Anal.

[25] Simon JL, Thompson RH (2006). The effects of undergarment type on the urinary continence of toddlers. J Appl Behav Anal.

[26] van Dommelen P, Kamphuis M, van Leerdam FJ, de Wilde JA, Rijpstra A, Campagne AE, Verkerk PH (2009). The short- and long-term effects of simple behavioral interventions for nocturnal enuresis in young children: a randomized controlled trial. J Pediatr.

[27] Caldwell PH, Codarini M, Stewart F, Hahn D, Sureshkumar P (2020) Alarm interventions for nocturnal enuresis in children. Cochrane Database Syst Rev 5:Cd002911. PMID: 32364251, PMCID: PMC7197139. 10.1002/14651858.CD002911.pub3 10.1002/14651858.CD002911.pub3 PMC7197139 32364251

[28] Glazener CM, Evans JH (2002) Desmopressin for nocturnal enuresis in children. Cochrane Database Syst Rev Cd002112. PMID: 12137645. 10.1002/14651858.CD002112 10.1002/14651858.CD002112 12137645

[29] Dhondt K, Van Herzeele C, Roels SP, Raes A, Groen LA, Hoebeke P, Walle JV (2015). Sleep fragmentation and periodic limb movements in children with monosymptomatic nocturnal enuresis and polyuria. Pediatr Nephrol.

[30] Ritvo ER, Ornitz EM, Gottlieb F, Poussaint AF, Maron BJ, Ditman KS, Blinn KA (1969). Arousal and nonarousal enuretic events. Am J Psychiatry.

[31] Forsythe WI, Redmond A (1974). Enuresis and spontaneous cure rate. Study of 1129 enuretis. Arch Dis Child.

[32] Yeung CK, Sreedhar B, Sihoe JD, Sit FK, Lau J (2006). Differences in characteristics of nocturnal enuresis between children and adolescents: a critical appraisal from a large epidemiological study. BJU Int.

[33] Larsson J, Borgström M, Karanikas B, Nevéus T (2023). Can enuresis alarm therapy be managed by the families without the support of a nurse? A prospective study of a real-world sample. Acta Paediatr.

[34] Breinbjerg A, Kamperis K, Jørgensen C, Rittig S (2022) Management of bedwetting - a survey among health care professionals. Paper presented at ICCS2022. Taipei Taiwan

[35] Hagstroem S, Borch L, Thorsteinsson K, Nielsen B, Kamperis K, Chai Q, Jørgensen C, Breinbjerg A, Borg B, Raaberg E (2023) ENURESIS ALARM FOR TREATMENT OF URINARY INCONTINENCE IN CHILDREN WITH COMBINED DAYTIME INCONTINENCE AND ENURESIS (THE ABDE-STUDY).Paper presented at ICCS 2023. Bahia, Brazil

