# Supplementary material for: Discontinuing absorbent pants in children with bedwetting: a randomized controlled trial
Source: Eur J Pediatr. 2024 Mar 12;183(5):2443–53. doi: 10.1007/s00431-024-05502-w (PMC11035460; doi:10.1007/s00431-024-05502-w)
Supplement: Supplementary file 1 — Supplementary file1 (DOCX 37 KB) [file 431_2024_5502_MOESM1_ESM.docx]

**Supplemental Information**

**Discontinuing Absorbent Pants in Children with Bedwetting: A Randomized Controlled Trial**

*Anders Breinbjerg, MD^1,2,*^; Konstantinos Kamperis, PhD^1,2^; Kristina Thorsteinsson^3^, MD; Cecilie Siggaard Jørgensen, PhD^1,2^; Juliette Rayner^4^, Jin Zhang^5^; Debora Garcia Rodrigues^5^; Luise Borch^6^, PhD; Prof Søren Hagstrøm^3^, PhD; Prof Serdar Tekgül^7^; Lien Dossche, PhD^8^; Prof Johan Vande Walle, PhD^8^; Prof Søren Rittig, DMSc^1,2^*

**Affiliations**

^1^Department of Pediatrics, Aarhus University Hospital, Aarhus, Denmark

^2^Department of Clinical Medicine, Aarhus University, Aarhus, Denmark

^3^Department of Pediatrics and Adolescent Medicine, Aalborg University Hospital, Aalborg, Denmark

^4^ERIC, The Children’s Bowel and Bladder Charity, 36 Old School House, Kingswood Foundation, Brittania Rd, Bristol, BS15 8DB, UK

^5^Global Product Safety, Stewardship & Medical Affairs, Kimberly-Clark Corporation, Tadworth, UK

^6^Department of Pediatric and Adolescent Medicine, Gødstrup Hospital, Herning, Denmark and NIDO, Centre for Research and Education, Gødstrup Hospital, Herning, Denmark

^7^Division of Pediatric Urology, Department of Urology, Medical School, Hacettepe University, Ankara, Turkey

^8^Department of Pediatric Nephrology, ERKNET center, Ghent University Hospital, Ghent, Belgium

***Corresponding author**

Anders Breinbjerg

Palle Juul-Jensens blvd. 99

8200 Aarhus N

[andbre@clin.au.dk](mailto:andbre@clin.au.dk)

+4522809382

**Supplementary text**

*Demographics*

At enrollment all children were measured in terms of height (cm) and weight (kg). A body mass index (BMI) was calculated (defined as weight in kg divided by the square of height in meters). Results can be found in **Supplementary Table 1**.

*Locations*

The study was conducted in seven different sites. In Denmark the centers consisted of the Department of Pediatrics, Aarhus University Hospital, Aarhus, Department of Pediatrics and Adolescent Medicine, Aalborg University Hospital, Aalborg, and Department of Pediatric and Adolescent Medicine, Gødstrup Hospital, Herning. In the UK enrollment was performed in ERIC, The Children’s Bowel and Bladder Charity, 36 Old School House, Kingswood Foundation, Brittania Rd, Bristol, BS15 8DB. In Belgium enrollment was done in the Department of Pediatric Nephrology, ERKNET center, Ghent University Hospital, Ghent.

*Ethics approvals*

The study was approved by the local ethical committees in Denmark (The Central Jutland Regional Committee on Health Research Ethics), Belgium (Ethisch Comité UZ Gent), and the United Kingdom (Northeast - Newcastle & North Tyneside 1 Research Ethics Committee NHSBT Newcastle).

*Absorbent pyjama pants vs. diapers*

APP differ from nappies or diapers in that they are designed with clothing-like materials/graphics and high absorbency, to provide the experience of an underwear-like garment in addition to protection from leakage.

*Sample size calculation*

We aimed to evaluate number of wet nights between the groups, setting the power to 99%, and the expected proportions for wet nights (margin was set to 2/7 wet nights) at the end of intervention/extension to 0.95 and 0.80 for continuing and discontinuing use of absorbent pants, respectively. As the children were randomized in a 2:1 ratio, we needed 15 children in the control arm to obtain a power of 99%. Due to the randomization ratio, that implicated a minimum number of 45 participants needed for randomization, and 15 were added to compensate for dropouts. This leaved a number of 60 children needed to obtain the power aimed for. No interim analysis was planned or conducted.

*Randomization*

Participants were randomly allocated using permuted block randomization in a 2:1 group allocation for discontinuation or continuation of the use of absorbent pants. The randomization was performed by an Interactive Web Response System (IWRS), a build-in feature in the EDC. Investigators or delegates would log onto the EDC and confirm the patient’s eligibility, and then proceed with the randomization.

*Statistical methods*

Primary outcome was analysed comparing the 95% CI for the difference between the means of the two study groups, with the predefined margin of 2/7 nights, comparing the last 7 days of intervention. The distribution of continuous data was assessed visually by drawing histograms, probability plots, and quantile-quantile plots. Furthermore, using the build in procedure of the SAS statistical software 9.4 (proc UNIVARIATE with the option: normal) test for normality was performed. Descriptive analyses were performed on the screened and the randomized participants to gain knowledge and characteristics of the included individuals. Continuous data are reported as mean with standard deviation (SD) or 95% confidence interval (CI); categorical values were summarized as number and proportion of the total study population. Descriptive analyses of the different sets of participants were prespecified.

Due to skewed dropout between groups and missing data, post-hoc analysis was planned. Four different sensitivity analyses were performed, including 1) imputing all missing data as wet nights, 2) imputing all missing data as dry nights, and 3) imputing all missing data drawing random numbers from a Bernoulli distribution with probability p, where p is the number of wet nights divided by number of non-missing data for that subject. p is assumed to be 0.5 if the number of non-missing data is less than 3 and all are wet nights, and 4) an intention-to-treat analysis including all randomized participants, imputing all individuals who dropped out as being wet 7/7 in the last week, and using the dataset from sensitivity analysis 3. Estimated marginal means are presented as effect measure for these analyses.

The difference between groups was estimated with a risk difference and an estimate of wet nights difference, relying on logistic regression. Despite not completing the core period, participants were included in the primary endpoint analysis if they had registrations for ≥21 days in the intervention period and no major violations of the protocol. The sensitivity analyses were carried out as described by Jakobsen *et al.*[1]. We have tested best-worst and worst-best case scenarios to show the range of uncertainty due to missing data. Since our sensitivity analysis 1 (all missing nights imputed as wet) and 2 (all missing nights imputed as dry) are uniform in their findings (same conclusion) we know that our results are not affected by missingness, and no systematic assessment of missingness (missing at random [MAR] or completely at random [MCAR]) was necessary.

Furthermore, participants were grouped in terms of full response (100% reduction in wet nights), partial response (50-99% reduction in wet nights) and no response (<50% reduction in wet nights), as according to the International Children’s Continence Society (ICCS) recommandations[2]. Data from the imputed sensitivity analysis 3 was used for grouping into response groups. Data from both core and extension period was used. To be eligible for analysis, non-missing data should be 4 nights or above in the last 7 days, along with a follow up of ≥21 days in the intervention period, and no major violations of the protocol. The chi^2^-test was used to compare groups.

Time-to-response in the two groups and time-to-drop-out were visualised using a time-to-event plot, including all data from the intervention period. Predictive factors of effect were sought through multiple logistic regression, using the covariates sex, age, and body mass index (BMI).

For the secondary endpoints frequency distribution and descriptive statistics were presented by group at randomization, after core period, and after extension period. Direct comparison between groups will be presented, but due to skewed drop out between the groups as described above, post-hoc analyses were performed as well, comparing non-responders in the no pants group with the pants group at the end of core period, to compare two groups comparable on effect, and only differentiated by the intervention.

A mixed model for repeated measures (MMRM) was fitted when applicable, including visit 2 scores, randomization group, visit month, and an interaction term between the randomization group and the visit month as covariates. A least square mean difference with standard error was estimated with this method, in participants with data from both visit 2, visit 3, and visit 4.

Individual risk factors (sex, height, weight, BMI) were investigated in a logistic regression model adjusting for the subject-id as clusters. In case of categorical risk factor such as sex, the probability was calculated for each combination of group and sex and were compared using odds ratio. In case of continuous risk factors such as age, odds ratio for each group was estimated and compared using odds ratio. The risk factors were further investigated in a multivariate logistic regression. No significant associations were established in either of the logistic or the multivariate logistic regression. See **Supplementary Table 2** for results on the multivariate logistic regression.

Baseline data for the 15 participants who dropped out during the core period in the no pants group are compared to the 53 participants who completed the core period in the no pants group (see **Supplementary Table 3**). No difference is seen between the groups in terms of age, height, weight, or BMI. Interestingly, it seems that boys tend to have a higher risk of early discontinuation.

**Supplementary Table 1**

| **Demographics for children randomized in the DryNites study, n = 105** | | | |
| --- | --- | --- | --- |
|  | **No-pants group, n = 70** | **Pants group, n = 35** | ***p-value*** |
| Age at inclusion, (years, mean [SD]) | 5.6 (1.09) | 5.4 (1.22) | 0.39 |
| Age range (years) | 4-8 | 4-8 | NA |
| Sex, female/male | 29/41 | 14/21 | 0.88^€^ |
| Weight (kg), mean (SD)* | 22.1 (3.58) | 23.9 (5.45) | 0.077 |
| Height (cm), mean (SD)* | 117.7 (7.21) | 118.2 (10.7) | 0.80 |
| BMI, mean (SD)* | 15.9 (1.64) | 16.9 (1.80) | 0.014 |
| BMI categories (% of sample)*^,$^ | Underweight: 5 (9.1)  Normal: 43 (78.2)  Overweight: 5 (9.1)  Obese: 2 (3.6) | Underweight: 0 (0)  Normal: 18 (66.7)  Overweight: 4 (14.8)  Obese: 5 (18.5) | 0.103^€^ |

*As one centre included several participants remotely, values for body metrics are based on n = 55 in the no-pants group, and n = 27 in the pants group.

^$^ Sex-specific BMI calculation related to age group performed. Underweight (<5th percentile for sex and age), Normal (from 5th to less than 85th percentile for sex and age), Overweight (from 85th to less than the 95th percentile for sex and age), Obese (≥ 95th percentile for sex and age).

^€^Chi-square test used for p-value calculation.

SD: standard deviation; BMI: body mass index; kg: kilogram; cm: centimeters.

**Supplementary Table 2**

| **Multivariate logistic regression model of potential risk factors of effect** | | | | |
| --- | --- | --- | --- | --- |
|  | **Estimate** | **Std. Error** | **Z-score** | ***p-*value** |
| Age (years) | -0.14 | 0.30 | -0.46 | 0.64 |
| Height (cm) | 0.11 | 0.17 | 0.65 | 0.51 |
| Weight (kg) | -0.27 | 0.42 | -0.65 | 0.51 |
| BMI | 0.62 | 0.61 | 1.01 | 0.31 |

BMI: body mass index

**Supplementary Table 3**

| **Comparison of baseline characteristics between drop outs and completers in the no pants group** | | | |
| --- | --- | --- | --- |
|  | **Drop outs, n = 15** | **Completers, n = 53** | ***p-value*** |
| Age at inclusion, (years, mean [SD]) | 6.1 (1.06) | 6.1 (1.09) | 0.88 |
| Sex, female/male | 2/13 | 26/27 | 0.018^€^ |
| Weight (kg), mean (SD)* | 21.3 (2.98) | 22.4 (3.79) | 0.34 |
| Height (cm), mean (SD)* | 117.0 (5.48) | 118.0 (7.86) | 0.56 |
| BMI, mean (SD)* | 15.6 (1.41) | 16.0 (1.73) | 0.43 |

^€^Chi-square test used for p-value calculation.

SD: standard deviation; BMI: body mass index; kg: kilogram; cm: centimeters.

**Supplementary References**

1. Jakobsen JC, Gluud C, Wetterslev J, Winkel P (2017) When and how should multiple imputation be used for handling missing data in randomised clinical trials - a practical guide with flowcharts. BMC Med Res Methodol 17:162

2. Nevéus T, Fonseca E, Franco I, Kawauchi A, Kovacevic L, Nieuwhof-Leppink A, Raes A, Tekgül S, Yang SS, Rittig S (2020) Management and treatment of nocturnal enuresis-an updated standardization document from the International Children's Continence Society. J Pediatr Urol 16:10-19
